# Cognitive training modifies disease symptoms in a mouse model of Huntington's disease

**DOI:** 10.1016/j.expneurol.2016.05.008

**Published:** 2016-08

**Authors:** Emma Yhnell, Mariah J. Lelos, Stephen B. Dunnett, Simon P. Brooks

**Affiliations:** The Brain Repair Group, Cardiff University School of Biosciences, The Sir Martin Evans Building, Museum Avenue, Cardiff, Wales CF10 3AX, United Kingdom

**Keywords:** Huntington's disease, Cognitive training, Mouse model, Operant testing, The 5-choice serial reaction time task

## Abstract

Huntington's disease (HD) is an incurable neurodegenerative disorder which causes a triad of motor, cognitive and psychiatric disturbances. Cognitive disruptions are a core feature of the disease, which significantly affect daily activities and quality of life, therefore cognitive training interventions present an exciting therapeutic intervention possibility for HD. We aimed to determine if specific cognitive training, in an operant task of attention, modifies the subsequent behavioural and neuropathological phenotype of the Hdh^Q111^ mouse model of HD.

Three testing groups comprising both Hdh^Q111^ mice and wildtype controls were used. The first group received cognitive training in an operant task of attention at 4 months of age. The second group received cognitive training in a comparable non-attentional operant task at 4 months of age, and the third group were control animals that did not receive cognitive training. All groups were then tested in an operant task of attention at 12 months of age. Relative to naïve untrained mice, both wildtype and Hdh^Q111^ mice that received cognitive training in the operant task of attention demonstrated an increased number of trials initiated, greater accuracy, and fewer ‘time out’ errors. A specific improvement in response time performance was observed in Hdh^Q111^ mice, relative to naïve untrained Hdh^Q111^ mice. Relative to the group that received comparable training in a non-attentional task, both wildtype and Hdh^Q111^ mice that received attentional training demonstrated superior accuracy in the task and made fewer ‘time out’ errors. Despite significant behavioural change, in both wildtype and Hdh^Q111^ mice that had received cognitive training, no significant changes in neuropathology were observed between any of the testing groups. These results demonstrate that attentional cognitive training implemented at a young age significantly improves attentional performance, at an older age, in both wildtype and Hdh^Q111^ mice. Attentional cognitive training also improved motor performance in Hdh^Q111^ mice, thus leading to the conclusion that cognitive training can improve disease symptoms in a mouse model of HD.

## Highlights

• Cognitive training improves HD related behaviour in HD knock-in mice.
• Attentional cognitive training improves cognition in both HD and WT mice.
• Attentional cognitive training can improve cognitive and motor signs in HD mice.

## 1 Introduction

Huntington's disease (HD) is caused by a CAG repeat trinucleotide expansion within the first exon of the *huntingtin* gene (MacDonald et al., 1993), the disease predominantly affects the medium spiny neurons (MSNs) within the striatum (Reiner et al., 1988) and there are currently no disease-modifying treatments. HD causes a range of symptoms including motor, cognitive and psychiatric disturbances (Diamond et al., 1992, Duff et al., 2007, Paulsen et al., 2008, Tabrizi et al., 2009), which significantly affect daily activities, the ability to manage independently and quality of life, even during the early stages of the disease (Helder et al., 2001, Ready et al., 2008). Cognitive disruptions in HD have been well documented, and can include specific problems with attention, cognitive flexibility and memory (Butters et al., 1985, Lawrence et al., 1998a, Lawrence et al., 1999, Lemiere et al., 2004, Sprengelmeyer et al., 1995). Difficulty in sharing attention between more than one task has been demonstrated to be a specific and core deficit in HD (Delval et al., 2008, Thompson et al., 2010). Therefore, cognitive training interventions, which focus on improving executive function, specifically focusing on attention, offer a potentially exciting therapeutic intervention for neurodegenerative diseases including HD.

Previous studies in HD patients (Lawrence et al., 1998a, Lawrence et al., 1996, Lemiere et al., 2004) and HD knock-in mouse models (Trueman et al., 2007, Trueman et al., 2012) suggest that problems with attention are a specific early deficit within the progression of HD. Therefore, it may be the case that cognitive training on an attentional task, at a young age, improves subsequent attentional performance as the disease progresses. This hypothesis is further supported by human studies in healthy individuals that suggest repeatedly conducting a task that requires specific aspects of executive function can improve cognitive function as people age (Ball et al., 2002, Willis et al., 2006). Furthermore, cognitive training studies have been conducted in patients with other neurodegenerative diseases including Parkinson's disease (PD) (Milman et al., 2014, París et al., 2011, Sammer et al., 2006, Sinforiani et al., 2004) and Alzheimer's disease (AD) (Clare et al., 2003, Clare and Woods, 2004, Davis et al., 2001, Farina et al., 2002, Hofmann et al., 1996). These studies have demonstrated that cognitive training, specifically focused on tasks of executive function, can improve both cognitive and motor outcomes in PD and AD. However, using cognitive training as a non-pharmacological therapeutic intervention is yet to be applied to the HD patient population.

Therefore, in this study, firstly, we aimed to determine if cognitive training on an automated operant task of attention alters the subsequent behavioural or neuropathological phenotype demonstrated by comparing animals (both wildtype and Hdh^Q111/+^) that had received attentional cognitive training to naïve animals that had not received any cognitive training. We also sought to further distinguish the behavioural and neuropathological differences observed between different types of cognitive training by comparing animals that had received attentional cognitive training (both wildtype and Hdh^Q111/+^) to those that had received non-attentional cognitive training (both wildtype and Hdh^Q111/+^). Thus, we aimed to determine the effects of cognitive training in both wildtype animals and in the Hdh^Q111^ mouse model of HD prior to further studies to translate the hypothesis into the HD patient population.

## 2 Methods

### 2.1 Animals

Hdh^Q111/+^ knock-in mice (Jax®, Bar Harbour, Maine, U.S.A.) were bred inhouse on a C57BL/6J background. 21 Hdh^Q111/+^ animals (11 female, 10 male) and 22 wild type animals (13 female, 9 male) were used. Animals were genotyped by Laragen Inc. (Culver City, California, USA), Hdh^Q111/+^ animals contained an average CAG repeat length of 141 (range 134–149 repeats). Animals were split into three groups, containing both wildtype and Hdh^Q111/+^ mice (Table 1), and kept in a temperature controlled environment (21 °C ± 1 °C) on a 12:12 hour light/dark cycle (lights on 06.00–18.00). Animals had ad-libitum access to food, although a water restriction regime was imposed during behavioural testing. Experiments were conducted in accordance with the 2013 European Union Directive 2010/63/EU.

### 2.2 Operant apparatus

**Fig. 1:**
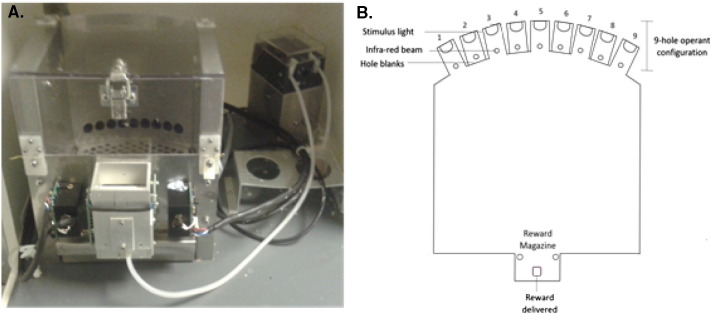
A. Picture of the mouse operant boxes used in behavioural testing. B. Schematic representation of the operant box apparatus, the back response wall contained 9 stimulus lights, 5 of which were used in operant testing. Holes 1, 3, 5, 7 and 9 contained stimulus lights and photocell detectors to detect nose pokes via breaking of an infrared beam, these correspond to holes A, B, C, D and E. Holes 2, 4, 6 and 8 were covered with well blanks. Each nose poke contained a stimulus light and an infrared beam to detect nose poke responses.

Operant testing was conducted in 16 9-hole operant boxes, controlled by a BehaviourNet Controller BNC MkII operating system (Campden Instruments, Loughborough, UK). Each operant box (Fig. 1A) was housed within a sound attenuating chamber, the rear wall was curved and contained a horizontal array of nine holes (11 mm diameter, placed 2 mm apart and 15 mm above floor level). Each hole contained a photocell infrared beam at the front to detect nose pokes. At the rear, a light emitting device (LED) provided the target visual stimulus. Only 5 of the 9 holes (termed A, B, C, D and E) were used in testing; black plastic film covers were inserted over unused holes. A peristaltic pump delivered strawberry milk (Yazoo®, Campina Ltd, Horsham, UK) to a magazine at the front of the box. Reward delivery was signalled by an LED above the magazine. Nose pokes into the magazine were detected via an infrared beam.

### 2.3 Water restriction regime

One week prior to operant training all animals were gradually water restricted, to a final restriction of 3 h of access to water per day, and introduced to Yazoo© strawberry milk reward, in their home cages. Animals received cognitive training 5 days per week and received daily access to water 4 h after operant training was completed. Ad-libitum access to water was given at weekends.

### 2.4 Experimental design

All operant training commenced at 4 months of age (Table 1). The first testing group (Group 1) received 15 days of nose poke training, followed by 20 days of training in the 5-choice serial reaction time task (5-CSRTT). The second group (Group 2) received a comparable total number of days (35 days) of nose poke training; however, for the final 20 days an extended nose poke training regime was used, as detailed below. The third testing group (Group 3) were controls and received no exposure to, or training in, the operant boxes; however they were handled and water restricted in the same way as the other testing groups.

### 2.5 Nose poke training

At 4 months of age, the animals in Groups 1 and 2 (Table 1) received 15 days of nose poke training. Training in responding into the magazine began with the delivery of 150 μl of strawberry milk into the magazine and illumination of the magazine light, after successful reward retrieval the magazine light was extinguished. The process was repeated for the 20 minute session time. Mice were then taught to poke on a simple fixed-ratio (FR1) schedule of reinforcement. The central hole in the array was illuminated and mice were required to respond to the stimulus by nose poking. A response to stimulus triggered the light to extinguish and the simultaneous illumination of the magazine light and delivery of 5 μl strawberry milkshake reward. The next trial was initiated with the illumination of the central light.

### 2.6 5 5-CSRTT operant training

After 15 days of nose poke training, Group 1 mice (see Section 2.5 and Table 1) were trained on the 5-CSRTT. Animals were trained to respond to a randomly-presented stimulus light, in order to receive a reward. After a correct response, the light was extinguished, 5 μl of milkshake reward was delivered, and the magazine light illuminated. Upon withdrawal from the magazine, the magazine light was extinguished and the next trial initiated. If a response was not executed within the stimulus length time, or the subsequent 10 s after the presentation of the stimulus (termed a ‘limited hold’), the light was extinguished and a ‘time-out’ period of 10 s was initiated by illumination of the house light. The process was repeated for the 30 minute session time. For the first 10 days, a 10 s stimulus was presented. A 2 s stimulus was used for the next 5 days and a 0.5 s stimulus was used for the final 5 days of testing.

### 2.7 Extended nose poke operant training

In addition to nose poke training, the animals in testing Group 2 received 20 days of extended nose poke training. The operant programme randomly selected a light stimulus which the animal was required to poke into, to obtain reward, for the duration of the 30 minute training session. The location of the response hole differed on subsequent days of training.

### 2.8 Operant testing

Operant testing was conducted in all groups at 12 months of age. All animals were gradually water restricted one week prior to operant testing and introduced to strawberry milkshake within their home cages. All animals were placed on the nose poke training programme, as described in Section 2.5. After the completion of training, animals were tested on the 5-CSRTT.

### 2.9 Tissue preparation and immunohistochemistry of free floating sections

Animals were culled via cervical dislocation, the brain was post fixed in 4% paraformaldehyde for 24 h and transferred into 25% sucrose in phosphate-buffered saline solution. Brains were frozen on a sledge-microtome (Leitz, Wetzlar), cut into 40 μm coronal sections, and collected in 12 parallel series. For immunohistochemistry, 1:12 series of sections were quenched for 5 min using 10% H_2_O_2_ (VWR, West Sussex, UK) and methanol (Sigma-Aldrich, Dorset, UK). Sections were blocked in 3% serum in Triton-X and Tris-buffered saline (TxTBS) for 1 h and incubated in a solution of TxTBS, 1% serum and primary antibodies raised against either DARPP-32 (AB_2314285, Cornell University, 1:30,000 (Ouimet et al., 1984), S830 (a gift from Gillian Bates King's College London, 1:25,000 (Sathasivam et al., 2001) or Neu-N (Millipore (MAB 377), 1:2000 (Mullen et al., 1992). Incubation in biotinylated-secondary antibody (1:200) was conducted for 2 h, then sections were incubated using an ABC kit (Vector Laboratories Ltd, Peterborough, Cambridgeshire) for 2 h.

Proteins were visualised using 3–3′-diaminobenzadine (DAB), before mounting on to double-subbed 1% gelatinised slides (Thermo Scientific, Menzel Gläser). After dehydration and delipidisation in 100% xylene, slides were coverslipped using DPX mountant (Thermo Scientific, Raymond Lamb, Leicestershire, UK).

For cresyl violet staining a 1:12 series of sections were mounted onto slides and allowed to air dry before incubation for 5 min in: 70% IMS, 95% IMS, 100% IMS, 50/50 chloroform alcohol, 95% IMS, 70% IMS, distilled water, cresyl violet solution and distilled water. Sections were dehydrated in 70% and 95% IMS, then destained by agitation in acid alcohol solution and coverslipped using DPX mountant.

### 2.10 Stereological analysis

Stereological quantification was conducted using Visiopharm Integrator System (VIS, version 4.4.6.9) software on an Olympus Canada Inc. Q-Imaging Microscope. For each slide, striatal sections were outlined under an × 1.25 objective lens. Defined striatal areas were then sampled in a systematically automated fashion and cells were counted using oil under the × 100 objective lens. The counting frame size used was 285 μm^2^. An Abercrombie correction factor was then applied to the total number of cells counted.

### 2.11 Statistical analysis

Statistical analyses were performed in IBM SPSS Statistic 20 software for windows. Repeated measures ANOVA tests, including the measures Group, Genotype, Stimulus duration and Hole, followed by simple effects analysis were conducted. This enabled both the overall effects of cognitive training and the effects of cognitive training on specific genotypes to be established. For histological analyses, univariate ANOVA tests followed by simple effects analysis were performed. Where significance was found *post-hoc* tests with Bonferroni corrections were applied to identify the locus of effects and their interaction(s). The significance level used throughout was α = 0.05.

## 3 Results

### 3.1 Operant training alters the ability to acquire the nose poke response

**Fig. 2:**
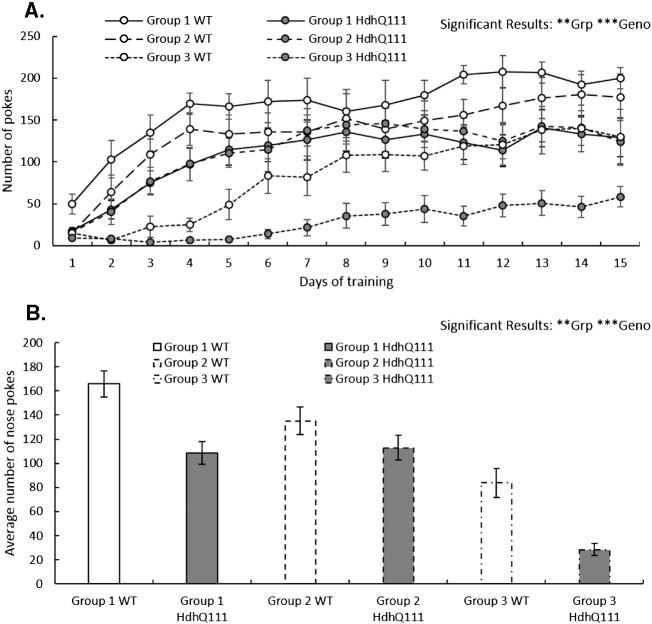
Acquisition of a nose poke response at 12 months of age. All animals began the testing phase of the experiment at 12 months of age, in order to learn how to response in the operant boxes all animals began the testing phase with learning the simple nose poke response. Group 1 received previous attentional cognitive training in the 5CSRTT. Group 2 had received comparable non-attentional cognitive training and Group 3 were naïve control animals. A. Acquisition of nose poke responses over 15 days of testing. B. Acquisition of nose pokes responses averaged over 15 days of testing. Error bars represent ± standard error of the mean. Significant results are indicated, Grp = Group, Geno = Genotype, no significant interaction effects were demonstrated. * p < 0.05, ** p < 0.01, *** p < 0.001.

Animals that received either cognitive training in the attentional operant task (Group 1) or comparable cognitive training in a non-attentional operant task (Group 2) were able to re-acquire the nose poke response rapidly, performing approximately 150 responses after 15 days of testing (Fig. 2A). However, the animals that were naive to the operant boxes (Group 3) took significantly longer to acquire the nose poke response than those that had previously been trained in the operant boxes (Group: F_2,36_ = 14.17, p < 0.01). After 15 days of testing the wildtype animals that were naive to the operant boxes (Group 3) were able to perform comparable levels of responses to Hdh^Q111/+^ animals that had previously received operant training (Groups 1 & 2), although they were not able to perform as many trials as wildtype animals that had received operant training. Hdh^Q111/+^ animals that were naive to the operant boxes were noticeably unable to initiate as many trials as the animals in the other testing groups. Overall Hdh^Q111/+^ mice performed fewer nose pokes than wildtype mice (Fig. 2B; Genotype: F_1,36_ = 17.57, p < 0.001), but there was no interaction between genotype and group (Genotype × Group; F_2,36_ = 0.84, p = n.s.).

### 3.2 Prior operant training in the 5-CSRTT modifies subsequent cognitive and motor behaviour in the 5-CSRTT

**Fig. 3:**
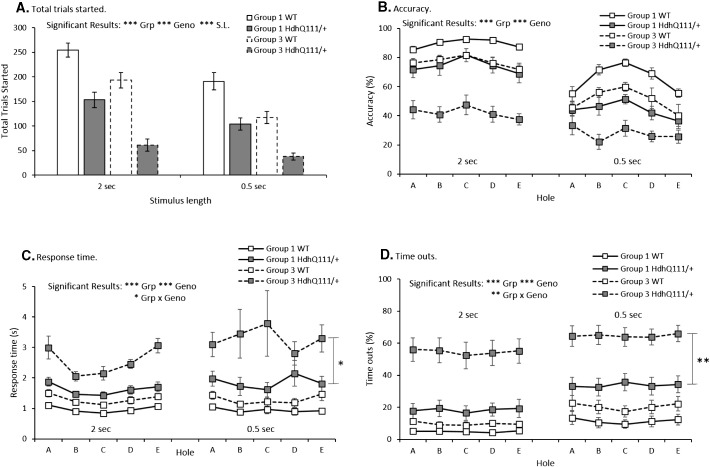
Attentional cognitive training improves subsequent performance in the 5-CSRTT. A. Total trials started, demonstrated that attentional cognitive training increases the number of trials started. B. Accuracy in responding was higher in the groups that had received previous training. C. Response time was faster in the groups that had received attentional cognitive training and significantly improved in Hdh^Q111/+^ animals that had received attentional cognitive training. D. Time outs made as a percentage of total responses were decreased in the group that received attentional cognitive training and significantly decreased in Hdh^Q111/+^ animals that had received attentional cognitive training. Data represents a total of 27 animals (Group 1 n = 14 (7 WT and 7 Hdh^Q111/+^) and group 3, n = 14 (7 WT and 6 Hdh^Q111/+^)). Data is shown for the final 5 days of testing at the 2 second and 0.5 second stimulus lengths. Error bars represent ± standard error of the mean. Significant results are indicated, Grp = Group, Geno = Genotype, S.L. = stimulus length. * p < 0.05, ** p < 0.01, *** p < 0.001.

Mice in Group 1 that received attentional cognitive training on the 5-CSRTT task at a young age initiated significantly more trials in the 5-CSRTT in comparison to Group 3 animals that had not received prior cognitive training in the task (Group: F_1,23_ = 21.83, p < 0.001), as seen in Fig. 3A. Hdh^Q111/+^ animals overall initiated fewer trials than wildtype animals (Fig. 3A, Genotype: F_1,23_ = 46.58, p < 0.001). Moreover, fewer trials were initiated when the shorter 0.5 second stimulus length was used, in comparison to the longer 2 second stimulus length (Fig. 3A, Stimulus length; F_1,23_ = 27.75, p < 0.001).

Despite an overall trend for Hdh^Q111/+^ animals to demonstrate a greater attentional deficit, in response accuracy, than wildtype animals, this trend failed to meet the threshold for conventional levels of significance (Fig. 3B, Genotype: F_1,23_ = 4.21, p = 0.052). Nevertheless, both wildtype and Hdh^Q111/+^ animals that received prior attentional cognitive training in the 5-CSRTT (Group 1) were significantly more accurate in responding in the 5-CSRTT than naïve animals (Group 3) (Fig. 3B, Group: F_1,23_ = 30.86, p < 0.001).

Animals in Group 1, which received attentional cognitive training demonstrated improved response times in comparison to naïve animals (Fig. 3C, Group: F_1,23_ = 37.36, p < 0.001). Hdh^Q111/+^ animals were overall significantly less accurate than wildtype animals (Fig. 3C, Genotype: F_1,23_ = 70.60, p < 0.001). Group 1 Hdh^Q111/+^ animals, that received attentional cognitive training, demonstrated significantly faster response times, in comparison to Group 3 Hdh^Q111/+^ animals that were naive the task (Fig. 3C, Group × Genotype: F_1,23_ = 6.41, p < 0.05). This pattern of results was also reflected in the number of time-outs made in the 5-CSRTT (Fig. 3D), animals that received attentional cognitive training made significantly fewer time outs in comparison to animals that were naïve to the task (Fig. 3D, Group; F_1,23_ = 17.76, p < 0.001). Hdh^Q111/+^ animals made significantly more time-out responses in comparison to wildtype animals (Fig. 3D, Genotype: F_1,23_ = 32.45, p < 0.001). Hdh^Q111/+^ animals made significantly fewer time outs in the task when they had received attentional cognitive training (Group 1), in comparison to Group 3 Hdh^Q111/+^ animals that were naïve to the task (Fig. 3D, Group × Genotype; F_1,23_ = 11.26, p < 0.01).

### 3.3 Does the operant training require an attentional component to modify subsequent behaviour in the 5-CSRTT?

**Fig. 4:**
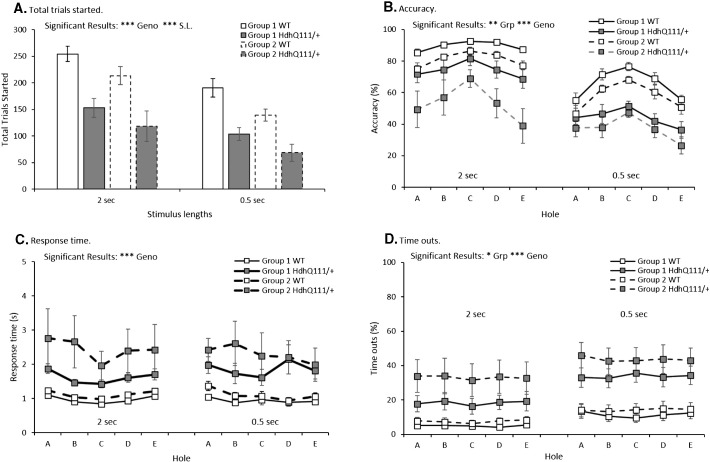
Comparison of attentional and non-attentional cognitive training on subsequent 5-CSRTT performance. A. Total trials started demonstrated less trials were made at the short stimulus length and by Hdh^Q111/+^ animals in comparison to wildtype animals. B. Accuracy in responding was improved in animals that had received attentional cognitive training in comparison to animals that had received cognitive training in a non-attentional task. C. Response time was decreased in Hdh^Q111/+^ animals but no effect of cognitive training regime was seen. D. Time out responses made. Despite a trend for Hdh^Q111/+^ animals that had received attentional cognitive training to make fewer time outs than Hdh^Q111/+^ animals that had received comparable non-attentional cognitive training, this interaction failed to meet significance. The data represents a total of 28 animals (Group 1 contained 14 animals, 7 of which were Hdh^Q111/+^ and Group 2 contained 14 animals, 6 of which were Hdh^Q111/+^). Error bars represent ± standard error of the mean.

Animals that received cognitive training in a non-attentional operant task (Group 2, Table 1) were able to initiate a similar number of trials in the 5-CSRTT as animals that had received attentional cognitive training in the 5-CSRTT (Fig. 4A, Group: F_1,24_ = 3.05, p = n.s.). Overall, Hdh^Q111/+^ animals initiated fewer trials in the 5-CSRTT than wildtype animals (Fig. 4A, Genotype: F_1,24_ = 40.94, p < 0.001) and fewer trials were initiated for all mice at the shorter 0.5 second stimulus length in comparison to the 2 second stimulus length (Fig. 4A, Stimulus length: F_1,24_ = 21.98, p < 0.001).

Interestingly, Group 1 mice that had received previous attentional cognitive training were significantly more accurate in the 5-CSRTT in comparison to animals that had received comparable cognitive training in a non-attentional task (Fig. 4B, Group: F_1,24_ = 8.84, p < 0.01). Although Hdh^Q111/+^ animals were less accurate overall than wildtype animals (Fig. 4B, Genotype: F_1,24_ = 18.16, p < 0.001), no significant interaction was demonstrated (Fig. 4B, Group × Genotype; F_1,24_ = 1.29, p = n.s.).

Despite a trend for mice that had received attentional cognitive training (Group 1) to demonstrate faster response times in the 5-CSRTT than mice that had received comparable cognitive training in a non-attentional task (Group 2), this trend failed to meet the threshold for statistical significance (Fig. 4C, Group: F_1,24_ = 3.61, p = 0.07). Furthermore, animals that received attentional cognitive training made significantly fewer time outs in the 5-CSRTT in comparison to animals that had received comparable cognitive training in a non-attentional task (Fig. 4D, Group: F_1,24_ = 13.57, p < 0.05). Hdh^Q111/+^ animals overall timed out more frequently than wildtype animals (Fig. 4D, Genotype: F_1,24_ = 19.84, p < 0.001): despite a trend for Hdh^Q111/+^ animals that had received attentional cognitive training to make fewer time outs than Hdh^Q111/+^ animals that had received comparable non-attentional cognitive training, this interaction failed to meet significance (Fig. 4D, Group × Genotype; F_1,24_ = 3.73, p = 0.065).

### 3.4 Does cognitive training modify the associated neuropathology observed in the Hdh^Q111/+^ mouse model of HD?

Stereological analysis conducted for S830, a marker of mutant huntingtin inclusions, showed that the number of cells affected by mutant huntingtin inclusions did not differ between Hdh^Q111/+^ animals that received attentional cognitive training (Group 1) relative to Hdh^Q111/+^ animals that received non-attentional cognitive training (Group 2) or naïve animals (Group 3) (Group: F_2,17_ = 1.24, p = n.s. data not shown). This effect was further reflected in cresyl violet, Neu-N and DARPP-32 cell counts, as no statistically significant effect of any cognitive training regime was observed between genotypes (cresyl violet: F_2,35_ = 1.58, p = n.s., Neu-N: F_2,33_ = 0.13, p = n.s., DARPP-32: F_2,36_ = 1.90, p = n.s., data not shown).

## 4 Discussion

The present study was designed to determine if cognitive training modifies subsequent behaviour or neuropathology in both wildtype mice and in the Hdh^Q111^ mouse model of HD. Animals that were given cognitive training in either an attentional or non-attentional task were significantly faster to reacquire the nose poke response, at 12 months of age, in comparison to naïve, untrained animals, and this was the case for both Hdh^Q111/+^ and wildtype animals. Nevertheless all groups reached stable levels of performance within approximately 15 days of testing. Moreover, animals that had received attentional cognitive training in the 5-CSRTT, demonstrated significant improvements in all behavioural measures of the 5-CSRTT when they were tested at an older age, and achieved higher levels of asymptotic performance than naïve, untrained mice. These improvements included: initiating more trials, making more accurate responses, executing faster responses and making significantly fewer time outs. Although there was a trend for attentional cognitive training to improve attentional performance in Hdh^Q111/+^ animals relative to naïve Hdh^Q111/+^ mice, this trend just failed to meet significance, due to the similar improvement demonstrated in wildtype animals that had received attentional training relative to naïve, untrained wildtype animals. However, a specific benefit of attentional cognitive training was seen in Hdh^Q111/+^ animals, as they responded significantly faster than naïve Hdh^Q111/+^ animals.

Animals that had received attentional cognitive training were more accurate and made fewer ‘time out’ responses than animals that had received comparable non-attentional cognitive training. There was also a trend for Hdh^Q111/+^ animals that had received attentional cognitive training to make fewer time outs than Hdh^Q111/+^ animals that had received non-attentional cognitive training, but this trend failed to meet conventional levels of significance. In comparison to animals that had received attentional cognitive training, non-attentionally trained animals, initiated the same number of trials and had similar response times.

While environmental enrichment has previously been used to improve cognitive function in HD mice (Nithianantharajah et al., 2008, Wood et al., 2010, Wood et al., 2011), using operant cognitive training to improve cognitive function had only previously been explored in the zQ175 HD mouse (Curtin et al., 2015). It has previously been demonstrated that cognitive training, at a young age, can attenuate some of the behavioural deficits observed in zQ175 mice. Thus, the positive effect of cognitive training, demonstrated in the present study in Hdh^Q111/+^ mice is supported by this previous study. In comparison to the previous study (Curtin et al., 2015), the present study has fewer animals and includes statistical analyses which consider numerous variables in the analyses, therefore the conclusions drawn can be considered robust. Furthermore, we have conducted further work in the present study, we have undertaken histological analyses and implemented specific cognitive training regimes (attentional and non-attentional) in order to determine the effects of cognitive training regimes that focused on different measures of executive function. Furthermore, the results observed are supported by previous studies in patients with other neurodegenerative diseases, which have found that cognitive training can improve both motor and cognitive disease symptoms in PD (Milman et al., 2014, París et al., 2011, Sammer et al., 2006, Sinforiani et al., 2004) and AD (Clare et al., 2003, Clare and Woods, 2004, Davis et al., 2001, Farina et al., 2002, Hofmann et al., 1996).

In the present study, Hdh^Q111/+^ animals that received attentional cognitive training, were shown to have improved response times in comparison to naïve animals, this may be reflective of an improvement in motor function or the ability to initiate motor function. Both Hdh^Q111/+^ and wild type animals that had received attentional cognitive training demonstrated improved attentional ability. These results are particularly relevant to consider with regard to the HD patient population, where attentional problems have been previously observed (Thompson et al., 2010). However, these results also have wide reaching implications for other neurodegenerative diseases and the aging population in general, as our study demonstrated that cognitive training, implemented at a young age, had significantly positive behavioural effects in wildtype mice. Cognitive training has been previously been demonstrated to prevent cognitive decline that would otherwise appear as part of the normal aging process in both mice (Forster et al., 1996, Gower and Lamberty, 1993) and humans (Ball et al., 2002, Hanninen et al., 1996, Levy, 1994, Persson et al., 2006, Schönknecht et al., 2014, Willis et al., 2006).

The specific type of cognitive training regime given to animals is an important factor in determining efficacy. While cognitive training in an attentional task at a young age, in the present study, significantly improved attention at an older age, comparable cognitive training in a non-attentional task did not improve attention at an older age. Thus, the attentional cognitive training given to animals demonstrated task specific improvements in cognitive function. In future iterations of this study it would be interesting to give attentional cognitive training in the 5-CSRTT of attention and then later test animals on a different behavioural task of attention to probe whether the training provides task specific benefits or whether the training can cause transfer effects to more produce general attentional benefits. However, the present study sought to determine proof of principle and included both attentional and non-attentional cognitive training to determine any differences in the type of cognitive training given. In the present study, equivalent motor training in a non-attentional task demonstrated that animals were able to initiate as many trials and respond as quickly in the 5-CSRTT as animals that had received cognitive and motor training in an attentional task. Therefore, although it can be concluded that the attentional cognitive training specifically improved attentional performance at a later age, equivalent motor training also improved other operant behaviours.

Furthermore, implementing attentional cognitive at a young age, has the potential to improve HD symptoms, in comparison to animals that are untrained, as was demonstrated in the improved response time performance of Hdh^Q111/+^ animals in the 5-CSRTT that received attentional cognitive training in comparison to naïve animals. This finding is of great clinical interest as it demonstrates that the HD brain may demonstrate a degree of plasticity, particularly if training can be implemented at a young age. Moreover, environmental enrichment activities such as exercise (which could be described as motor training) have previously shown benefit in animals models of HD (Harrison et al., 2013, Hockly et al., 2002, Pang et al., 2006) and HD patients (Busse et al., 2013, Busse and Rosser, 2007, Busse et al., 2008, Khalil et al., 2013, Khalil et al., 2012), although such exercise based therapies can be difficult to implement in this patient population and can lead to an increased risk of falls (Busse et al., 2009). Therefore, it may be the case that cognitive based intervention therapies would have a greater degree of acceptance and uptake by this patient population.

The 5-CSRTT was used in this case to measure attentional function in the Hdh^Q111/+^ mouse model of HD. Although attentional deficits have previously been shown in HD patients (Lawrence et al., 1998a, Lawrence et al., 1998b), more specific difficulties in sharing or distributing attention, often using dual tasks, has been shown to offer a more robust measure of determining attentional dysfunction in HD (Thompson et al., 2010). Therefore, in future manipulations of this study it may be useful to use a dual task as a measure of attentional dysfunction may produce even clearer and more definitive results.

Finally, immunohistochemical and stereological analyses were conducted to determine any gross neuropathological changes caused as a result of the cognitive training regimes. As the cognitive training intervention was relatively short (35 days), the lack of any significant differences between the numbers of S830 affected cells, cresyl violet, Neu-N or DARPP-32 stained cells between testing groups is perhaps unsurprising. However, it is likely that the neuronal connections underlying the observed behaviours were strengthened as a result of the cognitive training regimes. For example, molecular changes, such as increased levels of BDNF expression, have previously been found to rescue synaptic plasticity and memory in HD mice (Simmons et al., 2009) and rescue the HD phenotype (Giralt et al., 2011, Xie et al., 2010).

In conclusion, we demonstrate that cognitive training, implemented at a young age, can modify and improve behavioural symptoms in the Hdh^Q111/+^ mouse model of HD. The results are of potential clinical significance suggesting the possibility that cognitive training may be of therapeutic benefit for people with HD.

## Author roles

E.Y. study design, conducted experiment, conducted experimental analysis, and wrote manuscript. M.J. contributed to the study design, the experimental analysis of the results and revision of the manuscript. S.B.D co-supervised the project, contributed to the experimental analysis of the results and revision of the manuscript. S.P.B co-supervised the project and contributed to revision of the manuscript. All authors read the manuscript and approved it prior to submission.

## Figures and Tables

**Table 1:** Experimental design of animal testing groups. *An Hdh^Q111/+^ animal in testing group 3 became ill at approximately 8 months of age and was therefore culled reducing the number of Hdh^Q111/+^ animals in the testing group to 7. Abbreviation 5-CSRTT = 5-choice serial reaction time task.

	Training (4 months of age)	Testing (12 months of age)
Group 1 Attentional training (n = 14, 7 Hdh^Q111/+^)	Nose poke training (15 days) 5-CSRTT training (20 days)	Nose poke training (15 days) 5-CSRTT testing (20 days)
Group 2 Non-attentional training (n = 14, 6 Hdh^Q111/+^)	Nose poke training (15 days) Extended nose poke training (20 days)	Nose poke training (15 days) 5-CSRTT testing (20 days)
Group 3 Control (n = 15, 8 Hdh^Q111/+^*)	No training	Nose poke training (15 days) 5-CSRTT testing (20 days)
